# Time Management by Young People in Social Difficulties: Suggestions for Improving Their Life Trajectories

**DOI:** 10.3390/ijerph17239070

**Published:** 2020-12-04

**Authors:** Ángel De-Juanas, Francisco Javier García-Castilla, Diego Galán-Casado, Jorge Díaz-Esterri

**Affiliations:** 1 Department of Educational Theory and Social Pedagogy, Universidad Nacional de Educación a Distancia (UNED), 28038 Madrid, Spain; jordiaz@madrid.uned.es; 2 Department of Education, Universidad Camilo José Cela, 28692 Madrid, Spain; dagalan@ucjc.edu

**Keywords:** use of time, young people, social difficulties, leisure time, social work

## Abstract

This article covers the responses provided by professional practitioners in socio-educational intervention who are responsible for young people in social difficulties, in other words those facing personal and social issues that stop them from leading a normal life. It considers their suggestions for helping young people to better their lives by becoming autonomous, as well as to manage and use their time in their transition to adulthood. A qualitative study was conducted that used an open, ad-hoc questionnaire administered to thirty participants (Madrid, Spain), in which the data analysis involved MAXQDA Analytics Pro 2020 software. The results identify suggestions at macrosocial level targeting the system, legal status, therapy, safety nets, education and the range and provision of social services. On another level, suggestions for improvement were identified in an immediate setting in which the young people interact with agencies, practitioners and counsellors. An initial level featured mostly statements of support for autonomy from the system and social services. The second level contained mainly suggestions for agencies, centres and social services. The conclusion is that there are implications at different levels of social ecology according to Bronfenbrenner’s model (1994). The practical suggestions for young people’s self-sufficiency in the use and management of their time should therefore be flexible, linked and cater for their more therapeutic needs through to their leisure time.

## 1. Introduction

Young people’s time and their use of it should be understood as a vital process within their social development, and it may involve a raft of experiences (thinking, reflecting or socialising with friends, etc.) that are not always considered a topic of research [1]. This calls for their analysis as part of the society in which we live and interact today, characterised by immersion within a context of liquid modernity [2] in which everything is subject to rapid changes in both the real and virtual worlds [3], with technology dynamising communication and actions. This requires understanding the use of time from a holistic perspective that responds both to a cultural concept [4,5,6,7] and to the opportunities for its management of both an individual and collective nature [8]. Each of these factors may inform young people’s life narrative, with specific weight being attributed to myriad socialisations [9], schools, the media, etc. [10,11].

Young people take part in a broad range of experiences that impact upon their personal development, enabling them to achieve their goals [12,13] and, in turn, acquire a series of capabilities, skills and mindsets for laying the foundations for their adult lives [14]. In the event of social difficulties, the chances of properly using and managing time are minimised due to the limited possibilities for responding to existing demands [15] and to the subjective sense of discrimination that negatively affects them when taking part in social and cultural life [16,17,18]. The inequalities they face hold back the emergence of positive outcomes in their development [19,20], influenced by life experiences that prompt a lack of motivation, confidence and self-esteem [21,22,23]. This is due, above all, to risk factors [24,25] such as failure at school or even dropping out, disruptive family environments or the pernicious influence of their peer group [26,27,28,29,30], which trigger these situations of exclusion, discrimination and stigma [31,32]. Different studies have directly or indirectly addressed the inappropriate use of time. This may involve risk activities, often stemming from the need to generate certain emotions or feelings [33,34] or from boredom and dissatisfaction [35], where we encounter variables such as substance abuse, gambling or petty crime [36,37,38], triggering situations of deficiency or affecting certain vulnerable groups. Other more recent studies stress that those young people prone to boredom tend to use technology more, spend less time on pastimes and activities such as sport, drink alcohol more often (even becoming intoxicated) and run a greater risk of addiction to the Internet than those adolescents who do not suffer from boredom [39,40].

All these factors and circumstances mean that we need to stress the importance of socio-educational measures with young people in social difficulties as a way of improving their care and the processes designed to furnish the tools required for a successful transition. To do so, the first step involves the work being done by counsellors, practitioners and agencies, as a task that should consider the importance of support actions at schools or dynamic coordination with other institutions, as well as with the families themselves [41,42], where the professional role and its relationship with the recipients of the care is fundamental [43,44,45]. Educators in particular and agencies in general should be aware of their importance in the lives of those with limited personal and social resources [46]. This should be the first step to building a climate of trust [47,48] focusing on young people’s security and motivation toward time management in the different areas that make up their everyday lives, where confidence, active listening, emotional control, patience and effective support [49] are key features of this process.

It is also crucial to acknowledge these young people’s civic rights and duties, responding to their needs and expectations, whether inherent or emergent [11]. This means considering the need to involve this collective in different social mechanisms and activities that foster their active participation, enabling them to acquire social and cultural capital [50], emphasising their strengths [51] and adopting planning actions in their life projects [52,53], as a measure for tackling their disengagement due to a lack of trust in the system itself and the environment in which they live [54]. 

Finally, once the role of counsellors, practitioners and agencies has been addressed as parties responsible for improving care processes with vulnerable minors, we should not ignore the necessary involvement of officialdom for improving their care and intervention processes from a more global perspective. The transition to adulthood in itself is a delicate process [55], and the services this population receives often end when these individuals reach legal age [56]. Accordingly, besides improving their subsequent trajectories to ensure their transition to adulthood is successful, improvements and changes need to be introduced for reinforcing the care system [57], while at the same time implementing mechanisms designed to strengthen the associative fabric and socio-community networks, facilitate access to the labour market and guarantee that integral and plural approaches become the norm and are available through the multiple channels of communication that young people use today [58]. We therefore explore the specialised practices that could be introduced by those practitioners who counsel young people in social difficulty in order to help them manage and use their time, as well as the areas or aspects of their lives in which they can do so.

## 2. Materials and Methods 

This study’s overriding purpose was to investigate the responses of the counsellors and practitioners responsible for caring for, safeguarding, mentoring and educating young people in social difficulties regarding the specialised practical suggestions for change that can be introduced to help improve their management and use of time in their transition to adulthood. The young people receiving professional counselling who took part in our study are girls and boys aged between 12 and 21, living in sheltered accommodation, specific centres or at home with their families and who are exposed to situations of vulnerability. Furthermore, these young people attend specific centres or facilities to learn to live a healthier form of leisure and free time, thereby enabling them to alleviate the situation of social exclusion in which they are immersed. Moreover, this paper is the partial outcome of qualitative research into the time management of young people in social difficulties. The approach was inductive and not generalisable. Nevertheless, the aim was to achieve the greatest representativity possible during the recruitment of participants [59]. The technique of an open qualitative questionnaire was used [60] because it is a systematic method for gathering data from participants. This was followed by a content analysis of the professionals’ responses with no restriction of relevant categories established by the interpretation of the raw data. The entire research was based on the tenets of *Grounded Theory* according to Glasser and Strauss (1967) [61].

### 2.1. Participants

Intentional sampling was performed among the population of counsellors and practitioners dedicated to socio-educational intervention with young people in social difficulties in Madrid (Spain). This initially involved the careful identification and recruitment of ten key informants according to their possibilities of providing in-depth and detailed information on the research topic thanks to their professional condition, experience and responsibility. This first selection involved a selective sampling undertaken by the social agencies and institutions that took part in the study. Nonetheless, for the purpose of analysing the phenomenon in as much detail as possible, a second recruitment was made through snowball sampling in which the key informants led to the next participants and these to others, and so on successively. The essential criterion for participation in this second recruitment was that that the professionals had at least three years’ experience. The reasoning behind this sampling and which informed its strength lay in ensuring that the cases chosen would provide the greatest possible wealth of data, given that the main interest was not the metrics, but instead a thorough understanding of the phenomena and their latent social processes, through intensive and detailed knowledge based on the testimonies provided by counsellors and practitioners. This proactive or reasoned choice did not involve a specific prior number of informants, but instead the recruitment process ended once saturation had been reached and valuable and original information was available on the phenomenon under study.

The final sample consisted of thirty participants, of whom ten were male (33.3%) and twenty female (66.6%). The participants had a mean professional experience of twelve years and four months at state and private facilities. Most of them worked at youth centres and other socio-educational resources (N = 18, 60.1%), as well as at residential facilities (N = 8, 26.6%) and, finally, in social services for young people organised by local authorities (N = 4, 13.3%). In addition, most of them had higher education at least to degree level (N = 28, 93.3%); several of them also had a master’s degree (N = 8, 26.7%), and one had a PhD (3.3%). As regards their professional profiles, there were social educators (N = 11, 36.7%), social workers (N = 8, 26.7%), teaching assistants (N = 4, 13.3%), pedagogues (N = 4, 13.3%) and psychologists (N = 3, 10%). Each of these profiles contained at least one participant with duties in the management, administration and/or coordination of their workplaces. 

### 2.2. Instruments and Procedures

Use was made of an open, self-administered questionnaire that had been drafted ad hoc and was applied to the participants in an asynchronous manner via email [61]. Flexible use was made of this open questionnaire, rather than other data-gathering techniques, reducing the research outlay in terms of both funds and distribution times, as well as minimising the social desirability of the participants’ responses as the researchers were not physically present when they provided their answers [62,63]. Furthermore, this instrument minimised the order effect; paved the way for a decrease in social desirability by not influencing the participant’s thought process; and enabled practitioners to participate remotely in an asynchronous manner by fitting the data-gathering process into their own daily schedule.

Sociodemographic data were collected to characterise the sample. In addition, an extensive introduction to the research was provided together with a descriptive question designed to prompt ideas that would encourage the description of situations experienced, personal beliefs and other opinions on those specialised suggestions forwarded by the authorities, social services, agencies and the professionals themselves to improve the time management of young people in social difficulties as they transition into adulthood. Several people collaborating in the research process audited the questionnaire according to the intersubjective verification criterion [64]. The following question focused on the research purpose and was formulated in a clear, concise and direct manner: What specific improvements would you suggest for helping young people in social difficulties to manage and use their time, and in what areas or aspects of their lives?

The answers to this question were systematically processed by coding units of information in descending order that led to a level of descriptive coding and an axial one [60]. This involved producing a system of codes and subcodes that was consistent with the research purpose and allowed analysing the content of the answers to the questionnaire through a process of integration, creation and restructuring of categories consisting of two main codes and six subcodes (see Table 1).

The questionnaire was sent to the participants during the first quarter of 2020, together with information that introduced the research purpose and prompted the description of events, experiences and personal beliefs on the study topic. At the same time, an assurance of confidentiality and anonymity was provided in compliance with the ethical criteria of the Declaration of Helsinki (64th WMA General Assembly, Brazil, October 2013) together with the ethical approval of the Camilo José Cela University (code: 13_CEI_2020). After sending out the questionnaire, two specific dates for reminders were scheduled to complete the data-gathering process.

### 2.3. Data Analysis and Processing

Following Kuckartz and Rädiker (2019) [65], the first analysis codes were defined based on the open and inductive rating of the information units in the participants’ responses. Relationships were found between analysis codes through successive ratings, the names of the codes were refined, and the theory was constructed in a grounded manner. Data processing involved the software MAXQDA Analytics Pro 2020^©^ (VERBI Software GmbH, Berlin, Germany) version 20-0-8 for Mac OS. 

#### Consistency between Research Raters

Again, following Kuckartz and Rädiker for ensuring the stability of the data, the reliability of the category system was computed by measuring the agreement between raters. This involved six raters who were independent of the research and experts in the subject matter. The Kappa de Fleiss coefficient of consistency was calculated through the Real Statistic complement for the Excel software program in Office 365. The resulting coefficient was k = 0.743, which may be interpreted as a common coincidence in the identification of the codes on the units of information provided with a robust consistency strength [66]. 

## 3. Results

The number of statements compiled and the percentages obtained indicate that the participants made suggestions regarding time management and use with young people during their transition to adulthood in two major categories that were structured around two main levels or tiers: (1) influences from the immediate surroundings; and (2) macrosocial and contextual influences from the system and social services. The professionals provided more testimonies for the second level (34, 52.4%) compared to the first (30, 46.1%); in turn, only one participant in the study held the view that no improvements were needed to optimise the young people’s time management and use (see Table 2). 

From a more detailed perspective, regarding the professionals’ suggestions for young people’s time management and use in terms of the influence exerted on a macrosocial level and by the system and social services, more opinions were forthcoming on those improvement actions of a general nature for supporting autonomy from society, the system and social services (11, 32.4%), followed by those improvements in terms of care, safety nets and education (10, 29.4%), as well as those focusing on the access to, organisation and provision of services (10, 29.4%) and, finally, suggestions for improving the young people’s legal and administrative framework (3, 8.8%). 

As regards the testimonies on improvement suggestions at the particular level of the immediate environment, we find that the vast majority of the statements made by the professionals focus on measures that can be taken by agencies and centres working with the young people in support of their autonomy (21, 70%), compared to those suggestions that are related to the support and action of the professionals themselves (9, 30%).

**Figure 1:**
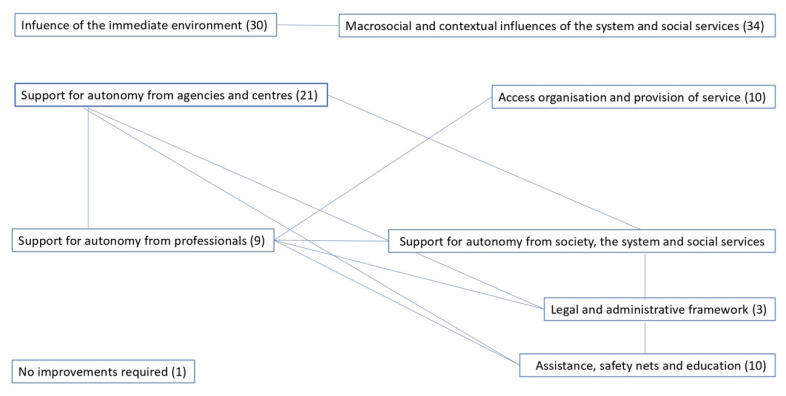
Relational model between codes and subcodes: suggestions for the management and use of time with young people during their transition to adulthood regarding levels of macrosocial influence, the system and social services, as well as the immediate environment. Source: authors’ own work.

Figure 1 contains a relational model of the data processed in the system of codes and subcodes used here.

### 3.1. Macrosocial and Contextual Influences of the System and Social Services

#### 3.1.1. Action in Support of Autonomy

Macrosocial actions and those specific to the system that enable young people to take control of their own lives through the management of their own time should be addressed from different standpoints, as expressed by this professional’s view:

“The system needs to consider awareness in general, empathising with the young person during their development, socio-labour integration, mental-affective care at this stage of their development, social skills, emotional intelligence, affective sexual education, family planning, subsidised housing…study and career guidance looking to the future…”

(Teaching assistant. C3.9-9)

This viewpoint is shared by other participants, as shown in the following response:

“It is crucial for the authorities to assign resources and programmes designed to fulfil this need for control over one’s own life, based on a personal life plan, supporting and mentoring decision-making, planning and managing times and tasks, motivation toward a purpose in life, striking the right balance between the time spent on duties, covering needs and free time for leisure…”

(Pedagogue. C14.9-9)

The suggested actions need to be flexible and focused on the inclusion of these young people within society:

“…it would be pertinent to establish more flexible and inclusive measures with activities that entail certain commitments and immediate rewards. The system for these young people is very rigid and often excludes them.”

(Social worker. C24.4-4)

Furthermore, these alternatives need to be diverse and provide a broad range of possibilities that meet these young people’s needs, as stated by the following professionals:

“There is a need to work on all the areas: family, school, professional, affective, emotional and social.”

(Social educator. C30.9-9)

“Providing a wide array of activities and instruction to suit their needs, tastes and interests, with them being the ones to manage, organise and plan them (participatory decision-making).”

(Pedagogue. C9.9-9)

“…this would lend prestige to the fabric of youth associations, reinforce it, and introduce a nationwide network of youth facilities that boost young people’s involvement in keeping with their interests. This would enable young people to take on responsibilities, and these would in turn teach them to organise their time.”

(Social workers. C12.9-9)

This involves fomenting the use of participatory ambits that foster healthy habits and behaviours:

“In terms of leisure time, this would provide an extensive programme of healthy behaviours through participatory workshops that include the proper, non-abusive use of ICTs, understanding this to be a necessary part of modern society, and fostering healthy leisure time through sport and personal development workshops.”

(Social Worker. C28.9-9)

#### 3.1.2. Legal and Administrative Framework

Concerning young people’s legal and administrative status, professional counsellors and practitioners refer to the difficulty this collective has to formalise it before they come of age, as noted by the following participant:

“They all complain that they don’t know what to do to regularise their administrative situation and they are forced to remain on hold without a job or any training whatsoever, with their only hope being to gain legal recognition before they come of age.”

(Pedagogue. C13.9-9)

Other views refer to the importance of reformulating the legal status of these young people once they find themselves within the system, especially when they come of age or reach their 21st birthday. A suggestion is on the table to extend the protection of this collective to the age of 23, rather than the current threshold of 21 in Spain, when the young people meet certain requirements within a structure created by the authorities:

“I would reformulate certain aspects of [Spain’s] Organic Law on the Protection of Minors, more specifically regarding the age at which that expires if no extension is assigned, extending it to a maximum age of 23. From the ages of 18 to 23, an individual would go from a Personal Education Project to a Project for the Transition to Adulthood, designed and supervises by the young person themself and an appropriate educator. This would mean that the assignation of resources would gradually be reduced and their nature would evolve. This transition would therefore receive guidance and support that would steadily be withdrawn as the individual grows in maturity and becomes self-reliant. This would require extending the network of resources for emancipation according to a structure orchestrated by the authorities.”

(Social educator. C22.9-9)

#### 3.1.3. Care, Safety Nets and Education

As regards the measures involved in care, safety nets and education, the practitioners affirm that advancing in such important issues as young people’s time use and management first requires improvements in the basic care provided for those in social difficulties:

“An important aspect involves catering for basic needs; they cannot progress unless they have their minimum requirements catered for (accommodation, maintenance…).”

(Social educator. C20.9-9)

The aim is to help all those young people according to their specific needs:

“…the main thing is that they reach the highest number of people that need support to cope with the transition in their own time and that they are aware of them.”

(Psychologist. C27.9-9)

One testimony highlights how important it is for the authorities to guarantee that young people will have somewhere to live once they are of legal age:

“ensuring that no young person is obliged or forced to become homeless or live on the street because they are of legal age, having to deal with solitude and with nothing to do during the day at such a young age.”

(Pedagogue. C9.9-9)

Other testimonies stress the importance of helping young people to use their time, ranging from official schemes for joining the labour market and training for a job:

“…another vital aspect involves employment. I don’t think a person can become fully independent and emancipated if they can’t find a proper job that gives them the stability to fend for themselves in an appropriate manner.”

(Social educator. C20.9-9)

Faced with this circumstance, the professionals make such suggestions as the following:

“I would suggest above all resources for finding employment through proper agreements with businesses so that these young people can regularise their situation if they need to, as well as the creation of a roadmap to self-sufficiency that can be effectively implemented through care resources while they are still minors, with programmes that prepare them for an independent life.”

(Social educator. C18.9-9)

“…create actions, activities, services in mentoring, training-employment, leisure…where they can gain a new perspective on the feasibility of their life plan and of their capabilities, being conscious of their possibilities and of the context’s limitations, learning to manage their time and tasks (youth, neighbourhood and community associations… foundations, council or regional services…).”

(Pedagogue. C14.9-9)

All this may include remedial socio-educational measures, as noted by the following professional:

“…as soon as possible include these minors in educational remedial classes or in specialist occupational modules that are more suited to each one’s expectations and tastes, and where at least some interest is generated in what they are doing.”

(Social worker. C28.9-9)

The aim is to proceed through the care and training provided within the framework of the community, as a safety net, and without ignoring the part families play:

“Promoting family schools, family guidance… within social services providing primary or specialised care, programmes organised by associations…”

(Pedagogue. C14.9-9)

#### 3.1.4. Access, Organisation and Provision of Services

As regards the promotion of improvements in terms of the organisation and provision of the resources that cater for these young people, the aim is

“…to provide both human and material facilities and resources, listening to their needs and expectations, in which they can develop their own initiatives.”

(Social educator. C4.9-9)

As well as:

“…provide the kids with a chance to contact expert professionals to help them at each stage of their process.”

(Teaching assistant. C16.9-9)

The professionalisation of human resources and their organisation is also a demand made by the professionals themselves:

“The first thing would be to introduce a more complete, professional and organised public service that would provide young people with support and a future”

(Teaching assistant. C1.9-9)

In turn, the professionals’ permanence in the same post could be considered for improving the organisation of resources:

“While the kids remain in the same facility for a long time, the professionals are constantly changing and they feel they don’t matter…”

(Teaching assistant. C16.9-9)

This organisation of resources calls for a more holistic approach in which public and private initiatives go hand-in-hand and are linked to each other:

“…this is crucial, linking all the public and/or private agencies to ensure close cooperation among all the necessary branches, not only in terms of leisure, but also regarding education, socio-health, culture, etc.”

(Teaching assistant. C1.9-9)

Specific suggestions are also made within the organisation of resources, such as this testimony by one of the professionals:

“Within the different areas of improvement, I would extrapolate different aspects of the intervention with the youngest individuals, looking for a change in different areas: engagement with the community and voluntary work, revealing and exploring attractive options for young people according to their current interests, as well as creating intergenerational programmes with other age groups (childhood, seniors…) and collectives…”

(Social worker. C17.9-9)

Within the educational environment, the suggestions are:

“…the incorporation of new professionals into the educational environment and collaborations with tutors and teaching staff; fostering personal autonomy and decision-making, also as mainstream goals during the development stages, creating projects and specific subjects in matters of everyday life (both basic activities in earlier stages, and advanced ones in more mature stages, handling of accounts, use of everyday tools, empowerment in household management…).”

(Social worker. C17.9-9)

### 3.2. Influences from the Immediate Environment

#### 3.2.1. Support for Autonomy from Agencies and Centres

This contains those improvement suggestions in the management and use of time by young people in social difficulties on a microlevel targeting the agencies and professionals working in them. We find that the suggestions geared towards agencies uphold the need to systemise and coordinate the outreach processes undertaken with these young people:

“A vital issue involves pursuing a proper outreach process and agreeing with the kid on the goals to be achieved, and with the educator’s help set timeframes for doing so…This is a major requirement, improving team coordination and the outreach and planning protocols.”

(Teaching assistant. C16.9-9)

There are suggestions that focus on developing young people’s autonomy through the management and use of study time and finding employment:

“Socio-educational intervention, making them see the benefits that study has for finding a job.”

(Social worker. C24.9-9)

“…concerning the time, they spend studying, training and learning, I would put in place a personalised intervention plan for each individual that would include the possible transfer to other schools in the network that are more suited to a given profile, as it is obvious that the current educational model is not much use.”

(Social workers. C28.9-9)

“I would work on such pillars as economics, self-sufficiency, personal growth, self-esteem, creativity, coeducation.”

(Social educator. C11.9-9)

“I would also introduce syllabuses or programming for the skills required for an independent life to be taught at the various facilities once the minor has reached the age of 17.”

(Social educator. C22.9-9)

Other suggestions refer to the implementation of mentoring actions involving the use of time for helping these young people to achieve a purpose in life:

“…there is a need to reinforce resources that address and impact upon the social and educational mentoring of these young people, in gaining awareness, the possibilities they have as individuals to make sensible use of their time in keeping with their preferences or goals.”

(Psychologist. C27.9-9)

“Time for himself. Freely chosen to respond to his hobbies, interests, motivations…which construct his identity.”

(Social worker. C24.9-9)

“…introduce educational programmes for the management of time and setting realistic goals.”

(Social worker. C23.9-9)

Elsewhere, there are suggestions on the management and use these young people make of their leisure and free time referring both to the use of technologies and to the performance of physical activity:

“Fostering leisure time that is constructive, fun, enjoyable and socially interactive by the agencies…”

(Social worker. C25.9-9)

“I would encourage the use of new technologies for time management (use of smartphones, email…). Workshops on time management adapted to the reality of today’s youth.”

(Social worker. C15.9-9)

“Acquisition of values and teaching life skills through physical activity.”

(Social worker. C24.9-9)

Finally, there are suggestions for the management and use of time that are related to the community, to a commitment to social and political engagement and to nurturing critical thinking:

“Time for taking part in collective projects in which their opinion counts, their interests are taken into account and they can play a part through the design of the search for answers…retaking initiatives in political or grassroots participation.”

(Social worker. C25.9-9)

“…working from the bottom up, dismantling gender stereotypes and deconstructing machismo in cultures; the relationship between action and reaction; that is, the consequences of your actions over the medium-to-long term, there would be a plethora of ways to focus this work starting from zero, besides the issue of gambling addiction, substance abuse, etc.”

(Social educator. C6.9-9)

#### 3.2.2. Support for Autonomy from Professionals

As regards the improvements suggested by the counsellors and practitioners, several testimonies mention the need to work on autonomy and time management in terms of immediacy until the young people focus on more medium-to-long term goals:

“The professionals have to prioritise and make the most of their demands for immediacy by helping them to reformulate them, while always listening or helping them to express their expectations regarding their occupational, social, and family lives, among others.”

(Psychologist. C27.9-9)

“…time management is important; having days in which the young people achieve their goals enables them to feel more comfortable with themselves and improve their self-esteem.”

(Social worker. C23.9-9)

There are also references to the importance of working on the young people’s control of their emotions and personal development:

“…it is essential for young people to properly control their emotions, being listened to and working on everything related to the areas of their personal competencies.”

(Social educator. C20.9-9)

“patience is another skill that has to be worked on…”

(Teaching assistant. C16.9-9)

“…a lot of work needs to be done on developing their capabilities and on helping them to find themselves and what they most need depending on their more immediate potential and motivation, all based on their actual social circumstances and the times we are living in.”

(Social worker. C28.9-9)

### 3.3. No Improvements Required

Finally, there was one testimony that considered that no improvements were required at any level, being expressed as follows:

“I don’t think any more improvements are needed, what is needed though is greater engagement by the young people in activities and resources.”

(Teaching assistant. C7.9-9)

## 4. Discussion

The starting point for our research involved exploring the responses of those professional practitioners in socio-educational intervention that are responsible for the care, safeguarding, mentoring and education of young people in social difficulties. The aim has been to compile improvement suggestions and reforms that they consider feasible for helping this collective to improve the management and use of their time during their transition to adulthood. The importance of this research is that it provides scientific support based on the professionals’ own personal testimonies and their experience with young people. This involves understanding their perspectives and their concerns regarding issues of such importance as the legal and administrative framework that has an impact on young people in social difficulties, their care, education and the safety nets in place within the system, the steps that could be undertaken by agencies, centres and the professionals working with them, etc. Accordingly, we trust that our findings will pave the way for the development of new and better practical suggestions that affect policies, social services, agencies, centres and the professionals themselves. 

During the coding of the information units of the participants’ responses, and while we were constructing the study’s grounded theory, we noted a certain similarity with Bronfenbrenner’s social ecological model (1994) [67], mainly because there were different suggestions that could basically be classified into two separate levels or tiers. A macrosocial level that refers to a broader section of society and affects policies, legislation and sundry systems that impact upon young people in social difficulties, but in which they are not directly involved. In turn, there is another tier or level that focuses on human development relationships that may correspond to a microsystem or immediate environment. Between these two levels there is obviously a third level or mesosystem that refers to the relationship between immediate environments and wider outside contexts specific to society at large. Our findings are consistent with those reported by Harder et al. (2020) [68], who used a review of principles for supporting practice in intervention and the care of children and young people to show that Bronfenbrenner’s ecological theory helps to explain how the research results have implications at different levels of social ecology. If the suggested changes are to be significant and improve practice, they need to be interconnected on the various levels.

Focusing more specifically on the views held by the professionals in our study, it is vital to encourage the meaningful community engagement of young people in social difficulties to ensure an effective transition process [69]. This process is understood to be an indicator of quality in interventions, as well as a platform for positive development, a means of achieving active citizenship [70], and young people’s right to take part in the decisions that affect their lives. Furthermore, the search for ambits of participation requires the commitment of the professionals [71], as they will be the key agents for achieving engagement and decision-making [72]. In addition, and besides the ambits of participation, we should not overlook the importance of fostering flexible and inclusive actions that underpin useful lifestyles, suited to the daily life of the child, adolescent or young adult, in order to prompt the assimilation of a normalised mode of behaviour that could have positive ramifications on a future independent life [47,73]. 

We should not forget that introducing concrete actions and fostering participatory ambits need to be accompanied by legal and administrative measures to favour effective adjustment to society. This leads us to call for a comprehensive care system that contains all the services that allow responding to needs in childhood and youth [74]. Moreover, the most extreme cases, namely, foreign minors, require a host society that is ready to accommodate the integration process [75] together with specific measures that do not abandon them once they reach the age stipulated by the Law on the Protection of Minors, and more so if we consider that their transition and subsequent trajectory cannot be understood without taking into account prior experiences and difficulties during the intervention [76], which generate their necessary support even though legislation determines the opposite. 

The professionals in the study also emphasised the importance of considering the needs of an especially vulnerable collective whose basic care is crucial. Instruction is one of the key components of those aspects with a particular need for attention, as it is important to remember that the most common factors used to characterise socially disengaged young people are related to an individual’s education and their job [77,78]. 

Cases of child poverty in the field of education increase the risk of “being left behind”; in other words, there is a high probability of becoming “disengaged” from learning processes that in modern democracies are considered to be a human right, thereby restricting their ability to keep up with their learning process and making it impossible for them to integrate at school [79]. This reality has also been reported by scholars such as Lacour and Tissington (2011) [80] and Reardon (2011) [81], who linked low income and processes informed by vulnerability with a lower academic performance and a greater chance of dropping out. In terms of employment, young people are more likely to be unemployed or on zero-hour contracts [82], but, if we add vulnerability to this mix, the difficulties are magnified considerably, increasing the risk of exclusion from the labour market that is often preceded by a record of school failure that hinders the processes of transition to employment [15]. 

Overcoming these difficulties requires support from officialdom, which should invest in these young people, roll out suitable schemes and policies and assign the appropriate resources for dealing with certain issues [83]. It is essential to ensure that young people at risk of social exclusion are in a position to build their own professional and social project in which actions and programmes are put in place [84] to increase this collective’s qualifications, as this is the point of departure for favouring employment and the exercise of citizenship [85]. 

Organisational improvements have also been highlighted by the professionals whose daily duties involve caring for young people in social difficulties. Job stability is one of those aspects that favour this organisational process, especially when we consider that many organisations render their services in facilities within the public domain or receive official funding through subsidies, whereby their jobs depend largely on the agency’s continuity in the management of specific resources [86]. Moreover, in this same vein, we should single out a study on the professional profile of educators at Centres for Minors in the Spanish region of the Community of Valencia, where some of the main aspects that are flagged as difficulties involve the terms and conditions of employment, highlighting factors such as precariousness or the scarce possibilities for in-house promotion [87]. Another key issue involves the provision of sufficient resources, as this will play a vital part in the success of interventions [40,88]. 

Finally, we should not forget the support for young people’s autonomy, as a process that should be reviewed regarding the work of both agencies and centres and the professional practice itself. In the first of these two cases, it is essential to know the minor properly, which means that agencies and centres need to design induction plans that favour the best and most appropriate intervention, ensuring that it caters for existing requirements at all times. Scholars such as Ávalos and El Homrani (2018) [89] stress the importance of a good needs’ diagnosis, besides flexible actions that allow modifications to be made based on an appropriate planning process. The young people’s own personal autonomy is also a key aspect that should be considered by the agencies and centres where these minors prepare to embark upon a fully inclusive process. This requires creating ambits and strategies for a successful transition, where it is essential for the young people to be capable of mastering the processes linked to autonomy, allowing decisions to be made in complex situations [90,91]. The autonomy process also involves proper time management, which should be based on three main pillars: (1) gratifying and healthy leisure activities, which may socially promote and safeguard vulnerable young people [92]; (2) mentoring based on a climate of trust and a suitable relationship between the professional and the recipient [44,48]; and (3) obtaining a young person’s engagement with the community, reinforcing the formation of values, setting guidelines and patterns of behaviour that contribute to the acquisition of competencies in relationships with others, beliefs and good habits for adopting planning actions in their life projects [40].

## 5. Conclusions

The professional practitioners’ responses and the number thereof show that the suggestions made can be grouped at both a macrosocial level, focusing on the system and officialdom, and at a microsocial level specific to the immediate environment involving agencies and professionals. Although there are more testimonies involving those suggestions that may be introduced at macrosocial level, there is only a small gap between them and the suggestions at the microsocial level. Likewise, within the macrosocial level, the professionals place greater emphasis on those suggestions designed to improve the control over their own lives of young people in social difficulties compared to other suggestions focusing on these young people’s care and training, organisational and provisioning aspects and their legal and administrative circumstances. The measures considered within each subcategory complement the information and ratify the professionals’ interest in addressing the time management and use of young people in social difficulties from different approaches (employment, emotional, affective-social control, etc.), with flexible and inclusive actions, catering for the young people’s needs in participatory ambits and according to the premise of fostering healthy lifestyle habits. In turn, there is a call for reformulating the legal and administrative framework to extend the young people’s protection to the age of 23 in those cases as deemed expedient. The professionals also see a need for improvements in basic care, especially in terms of guaranteeing shelter. Support should also be provided in remedial education and employment. While the organisation and provision of resources may also be subject to improvement, there are also suggestions for combining the efforts of public and private incentives, as well as providing facilities and resources to cater for these young people’s needs and expectations.

As regards the microsocial level involving the immediate environment, the professionals find more openings for improvement in the agencies and centres where they work. This is revealed by the large number of coded statements on this matter. The focus of the improvements for the management and use of time is on the need to systemise and coordinate the processes of care, the development of autonomy, employment and the ambit of leisure and free time, as well as different mentoring activities for young people in social difficulties. As regards the improvements corresponding to the actual work of the professionals themselves, there are testimonies on the need to focus young people’s attention on the achievement of medium- and long-term goals, as well as work on the control of emotions and personal development. 

All these suggestions, at one or another level or tier, are interconnected and should be applied from a joint perspective to introduce systematic changes that improve the inclusion processes of young people in social difficulties.

### Limitations and Implications

The research into improvement suggestions for making the best use and management of time and for autonomy in the transition to adulthood of young people in social difficulties through practitioners’ responses is a complex process. Our systemisation of the information uses direct, first-hand opinions, albeit adopting a selective stance that underpins the research and which, in turn, involves us as an active part of the study. This may be one of the research’s limitations. 

Although this study encompasses a large number of the replies provided by the professionals, the sample’s size and selection may also be considered a limitation. Nevertheless, key informants were involved that are elusive participants. Furthermore, future research could study the young people’s own views on these improvement suggestions related to their time management and use and their autonomy. This would provide a broader, more holistic and more complex perspective of the situation by those directly involved. Furthermore, our findings do not reveal major differences depending on the informants’ professional profiles. Nevertheless, future research may be designed to gather sufficient data for this purpose that reports conclusive findings in this matter. It is also deemed important to launch future studies for comparing the testimonies of these young people in social difficulties to the opinions of their peers in general. 

## Figures and Tables

**Table 1:** Codes and subcodes arising from the data analysis.

Codes	Subcodes
*Macrosocial and contextual influence of the system and social services* Testimonies on the improvement suggestions that could be implemented by society. Consideration should be given to the structure of social opportunities involved in the task that can be undertaken by the authorities and social services. Young people cannot be directly involved at this level, but they can perceive their influences in the interaction within a more immediate environment.	*Support for autonomy from society, the system and social services*. Improvement suggestions targeting the system and society from different levels and services. They focus on the inclusion of young people in society, they are flexible and involve a wide range of possible forms of participation.
	*Legal and administrative framework*. Improvement suggestions focusing on regularising the legal and administrative status of young people in social difficulties.
	*Care, safety nets and education*. Improvement suggestions designed to provide basic safety nets for young people, as well as their self-sufficiency through education, according to their specific needs.
	*Access, organisation and provision of services*. Improvement suggestions focusing on the arrangement and provision of different resources and services through different intervention areas.
*Influences of the immediate environment* Responses featuring those practical suggestions for improvement that may be undertaken from a tier or level that is closer to the young people and contains structures with which they have direct contact. These suggestions encompass relationships and interactions involving agencies, centres and services of social action, as well as the professionals of socio-educational intervention.	*Support for autonomy through agencies and centres*. Those suggestions that call for the need to systemise and coordinate care processes to help young people become autonomous, through mentoring and respect for each young person’s own identity.
	*Support for autonomy from professionals*. Those suggestions that refer to the need to work on time management for giving meaning to these young people’s lives. These suggestions include setting long-term goals and objectives, emotional control and personal development.
*No need for improvement suggestions*	--

Source: authors’ own work.

**Table 2:** Detail of contributions and percentages of suggestions on the management and use of time with young people during their transition to adulthood (frequency of contributions, N).

Codes	Subcodes	N	Per cent of the Code	Per cent of the Overall Figure
Macrosocial and contextual influences of the system and social services	Support for autonomy from society, the system and social services	11	32.4	17
	Legal and administrative framework	3	8.8	4.6
	Care, safety nets and education	10	29.4	15.4
	Access, organisation and provision of services	10	29.4	15.4
	Total	34		52.4
Influences from the immediate environment	Support for autonomy from agencies and centres	21	70	32.3
	Support for autonomy from professionals	9	30	13.8
	Total	30		46.1
No improvement suggestions required	--			1.5
	Total	1		
	Total	65	100	100

Source: authors’ own work.

## Data Availability

The datasets generated for this study are available on request to the corresponding author.
